# Primary Colorectal Lymphoma Presenting as a Perisigmoid Abscess: A Report of a Rare Case

**DOI:** 10.7759/cureus.87229

**Published:** 2025-07-03

**Authors:** Ana Rivera-Garcia Granados, Shadany J Flores-López, Juan A Rodríguez-Inurrigarro, Catalina Ortiz-Monasterio, Paulina P Rábago-Sánchez

**Affiliations:** 1 Colon and Rectum Section, Department of Surgery, Central Military Hospital, Mexico City, MEX; 2 Department of Surgery, ABC Medical Center, Mexico City, MEX

**Keywords:** abscess, colorectal neoplasms, non-hodgkin lymphoma, perisigmoid abscess, primary colon lymphoma

## Abstract

Primary colon lymphoma is an extremely rare condition that can affect men more frequently. It typically presents with symptoms such as abdominal pain, weight loss, and a change in bowel habits. In this case, we report on a 78-year-old female patient who visited the Emergency Department due to pain in the lower left quadrant of the abdomen. The initial evaluation included a computed tomography scan, which revealed concentric thickening of the sigmoid colon, associated with a mesenteric border abscess, as well as lymphadenopathies in the retroperitoneum and mesentery. These radiological findings raised suspicion of perforated diverticulitis, as well as lymphoma, as differential diagnoses. Antibiotic treatment was initiated to control the abscess, and a colonoscopy with biopsy was subsequently performed. Histopathological examination confirmed the diagnosis of B-cell non-Hodgkin lymphoma (NHL), classified as clinical stage IV due to the observed lymphatic dissemination. Although chemotherapy was offered as a potential treatment, the patient declined it for personal reasons. Primary colorectal lymphoma is a sporadic entity, and to the best of our knowledge, there are very few articles reporting it in association with a mesenteric abscess. This case highlights the importance of considering rare diagnoses in patients with gastrointestinal symptoms. Furthermore, it underscores the need for a multidisciplinary approach to managing such conditions, involving collaboration among radiologists, gastroenterologists, hematologists, oncologists, and pathologists to ensure accurate diagnosis and appropriate treatment.

## Introduction

Gastrointestinal lymphoma is the most common type of extranodal lymphoma; however, colorectal localization is rare, representing only 3% of cases [1,2]. It is the third most common type of colorectal neoplasm, following adenocarcinoma and neuroendocrine tumors, yet it accounts for less than 0.1% of these [3,4]. Most of the time, colorectal lymphoma presents with abdominal pain, weight loss, and a change in bowel habits, although presentations can vary. Therefore, the present article aims to explore a novel case of colorectal lymphoma accompanied by a perisigmoid abscess. We discuss the findings by elaborating on the clinical presentation, diagnosis, and treatment.

## Case presentation

A 78-year-old female with a history of systemic arterial hypertension and type 2 diabetes presented with left iliac fossa pain, accompanied by a two-day history of fever. Upon admission to the Emergency Department, the vital signs were within normal limits - with blood pressure (127/86 mmHg) and respiratory rate (18 breaths per minute) - except for her temperature (38.1°C) and heart rate (115 beats per minute). Physical examination revealed superficial and deep tenderness in the left iliac fossa and hypogastrium.

Laboratory tests showed leukocytosis (15,600 × 10³/μL), which was predominantly attributable to neutrophilia (78%). The entire list of laboratory results is presented in Table 1.

**Table 1:** Patient blood count

Parameter	Results	Reference Values
Hemoglobin	14.2 g/dL	11.2 - 17.5 g/dL
Hematocrit	43%	33 - 49%
Mean Corpuscular Volume	90 fL	80 - 100 fL
Mean Corpuscular Hemoglobin	30 pg	27 - 33 pg
Total White Blood Cells	15,600 /μL	4,500 - 11,000 /μL
Neutrophils	78%	40 - 70%
Lymphocytes	15%	20 - 45%
Platelets	280,000 /μL	150,000 - 450,000 /μL

**Figure 1:**
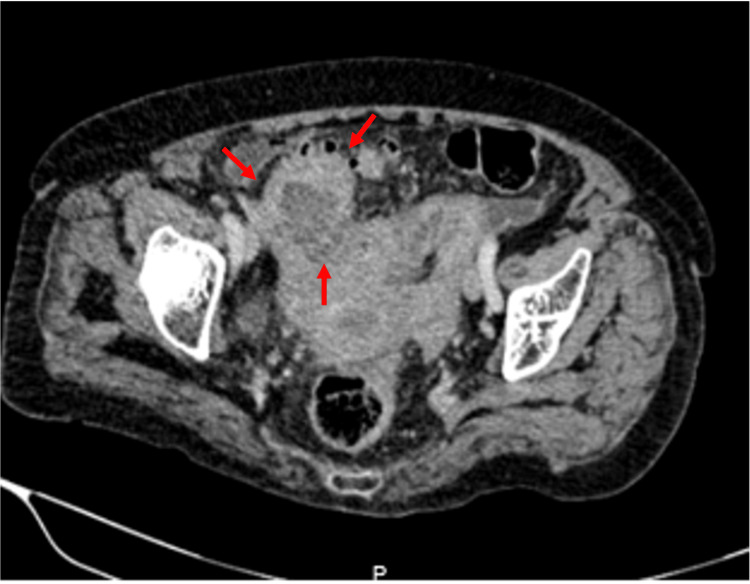
Axial view of the abdominal tomography The area indicated by red arrows shows the thickening of the sigmoid colon wall, associated with an abscess (approximately 79.3 mL in volume).

An abdominal computed tomography (CT) revealed sigmoid colon diverticulosis, as well as irregular, deforming, non-stenotic concentric wall thickening of the sigmoid colon up to the rectosigmoid junction, associated with an abscess (with semi-liquid content and multiloculated air corpuscles, approximately 79.3 mL in volume) on the mesenteric border, and necrotic lymph node conglomerates measuring 47 × 56 mm, as well as lymphadenopathies of 11 mm in the short axis in the sigmoid mesentery. Based on these CT findings, perforated diverticulitis as well as lymphoma were suspected as differential diagnoses. Given the presence of an abscess, antibiotic treatment was initiated to control the infection and reduce the patient's symptoms (Figure 1). Interventional radiology was consulted for abdominal collection drainage, but it was considered unsuitable due to an inadequate window.

**Figure 2:**
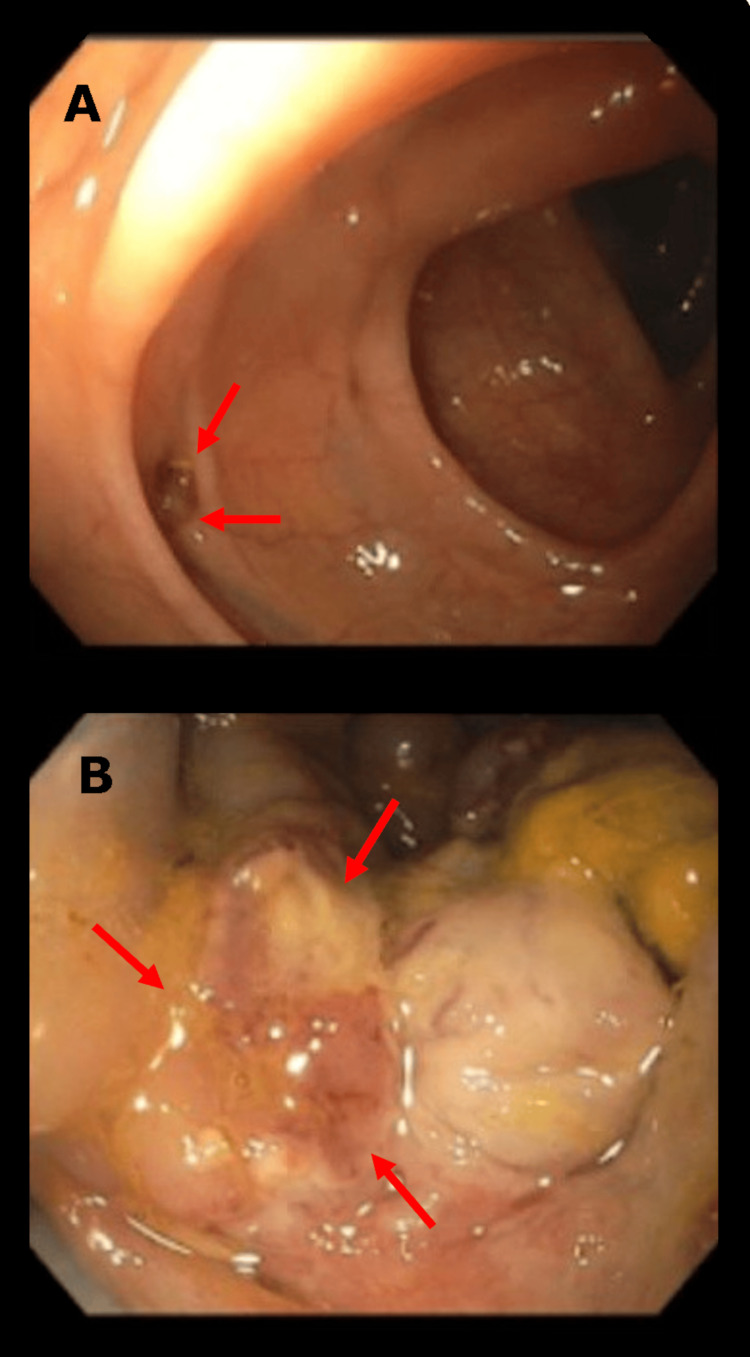
Colonoscopy findings A) Sigmoid colon showing a diverticulum without associated complications (arrows). It was located 55 cm downward from the infiltrative ulcer. The surrounding colonic tissue displays normal architecture. B) Excavated and infiltrative ulcer approximately 15 mm in size located in the ascending colon, with irregular borders and purulent exudate (arrows), consistent with tumoral infiltration.

After four days of intravenous antibiotic treatment based on ceftriaxone 1 g per day, a colonoscopy was performed, showing almost complete resolution of the abscess and revealing an ulceroinfiltrative lesion located between 12 and 19 cm from the anal verge, covering approximately 60% of the lumen. Another finding was the presence of a narrow-neck diverticulum with a clean base and no evidence of complications, located 55 cm from the infiltrative ulcer (Figure 2).

A biopsy was taken during colonoscopy, which revealed the presence of lymphoma with a histological subtype of diffuse large B-cell lymphoma. Immunohistochemical markers were positive for CD20+, BCL-6+, and CD45+.

As part of the staging, a fluorodeoxyglucose positron emission tomography revealed supra- and infra-diaphragmatic lymphadenopathies and a sigmoid parietal injury associated with hypermetabolism related to lymphoproliferative activity. Therefore, a hematologist was consulted to determine the definitive diagnosis, reporting stage IV diffuse large B-cell lymphoma attributable to disseminated extra-lymphatic tissue with associated lymph node involvement.

Two treatment options were proposed: chemotherapy based on the R-miniCHOP scheme, including rituximab, cyclophosphamide, doxorubicin, vincristine, and prednisone; and palliative chemotherapy, including rituximab, prednisone, non-steroidal anti-inflammatory drugs, and ondansetron. However, the patient and her family made a personal decision to decline these treatments, which must be respected in the context of patient autonomy.

## Discussion

Non-Hodgkin lymphoma (NHL) is a group of malignancies originating from B or T lymphocytes. They can present aggressively, with acute or subacute manifestations, rapid tumor growth, and associated B symptoms (fever, night sweats, and weight loss). High-grade lymphomas include diffuse large B-cell lymphoma, Burkitt lymphoma, and lymphoblastic lymphoma of T or B cells, among others [5]. Low-grade lymphomas generally have an indolent course, with progressive lymphadenopathy, hepatosplenomegaly, and pancytopenia, among other symptoms. These include follicular and marginal zone lymphomas [6].

Primary gastrointestinal lymphomas are rare, accounting for 5% of NHL. Only 10%-20% of this group are colorectal in location [4]. In 1961, Dawson et al. [7] published diagnostic criteria for primary colorectal lymphoma, which currently remain valid [8]. These are: (a) no superficial lymphadenopathy during the initial evaluation; (b) no evidence of mediastinal lymphadenopathy; (c) normal leukocyte count and routine bone marrow biopsy; (d) during laparotomy, only local nodal disease is present; and (e) liver and spleen are free of disease. However, alternative definitions for nodal and extranodal disease do exist. Sometimes, extranodal disease can spread and appear as nodal disease, making it difficult to distinguish between them in nearly 10% of patients. Krol et al. proposed a wider definition, considering primary extranodal lymphoma in all patients whose extranodal component is clinically dominant despite disseminated disease [9].

Primary colorectal lymphoma is more common in men, and although it can occur at any age, the average age of onset is 60 years [4,10,11]. The ileocecal region is the most common site of involvement among colorectal lymphomas, followed by the rectum. This may be due to this segment’s large amount of lymphoid tissue [12]. The most common presentation is lower gastrointestinal bleeding; however, patients may also experience abdominal pain, diarrhea, or intestinal obstruction (in cases of pain) [4,10]. Symptoms depend on the tumor’s location, which makes diagnosis challenging. In cases of ileocecal lymphoma, the most common symptom is weight loss, followed by melena or hematochezia [13]. In the current literature, to our knowledge, there is no evidence of colorectal lymphoma coexisting with an abscess; we found only one case of an abscessed colosplenic fistula [8].

Although the final diagnosis is histopathological, a combination of clinical presentations, imaging, and endoscopic findings is critical when diagnosing this entity. It presents as a long, circumferential thickening of the colonic wall. There may be aneurysmal dilation of the lumen, possibly due to the loss of the muscularis propria, and lymphoma's destruction of the autonomic nerve plexus [14].

Similarly, a colonoscopy will reveal a circumferential lesion. Sometimes, this lymphoma may present as a "smooth" thickening, and biopsies could not report any histopathological alteration, due to the fact that tumor infiltration might be submucosal. In addition, focal colorectal lymphoma may be present as polypoid or ulceroinfiltrative lesions [12].

The most common subtype of primary colon NHL is diffuse large B-cell lymphoma. Various risk factors for this type of NHL include hereditary immunodeficiencies, autoimmune diseases, immunosuppression, and viruses such as HIV, hepatitis C virus, herpesvirus 8, and Epstein-Barr virus [15]. Histopathologically, it is characterized by large lymphoid cells with a high nucleus-to-cytoplasm ratio, an irregular nucleus, and a prominent nucleolus with basophilic cytoplasm. These cells usually express markers such as CD19, CD20, CD22, and CD79a [15].

Due to its low incidence, treatment is not well defined. There is ongoing debate about chemotherapy alone, neoadjuvant chemotherapy followed by resective surgery, or surgery followed by chemotherapy [4]. Some studies report improved survival with surgery [16], while others suggest that surgery should be reserved for complications [17]. Cai et al. [18] determined that ideal candidates for surgery are those in early clinical stages with right colon involvement. When treating with chemotherapy, it is essential to consider that the combination of rituximab with cyclophosphamide, doxorubicin, vincristine, and prednisone (R-CHOP) has been associated with colonic perforation and the need for emergency surgery [4].

## Conclusions

Primary colorectal lymphoma is uncommon, and presentation with an abscess can pose a diagnostic challenge. A high index of suspicion is needed to avoid delayed diagnosis. This case underscores the importance of maintaining a broad differential when evaluating abdominal abscesses, particularly in elderly patients. Timely use of imaging, endoscopy, and histopathological assessment is crucial for accurate diagnosis. Given the rarity and lack of standardized treatment protocols, management should be individualized with input from a multidisciplinary team.
